# Antifungal Action of Metallic Nanoparticles Against Fungicide-Resistant Pathogens Causing Main Postharvest Lemon Diseases

**DOI:** 10.3390/jof10110782

**Published:** 2024-11-11

**Authors:** Carina G. Baigorria, Luciana Cerioni, Mario A. Debes, Ana E. Ledesma, Patricio Alastuey, Mónica Tirado, Sabrina I. Volentini, Viviana A. Rapisarda

**Affiliations:** 1 Instituto Superior de Investigaciones Biológicas (INSIBIO), CONICET-UNT, and Instituto de Química Biológica “Dr. Bernabé Bloj”, Facultad de Bioquímica, Química y Farmacia, UNT, San Miguel de Tucumán 4000, Argentina; 2 Cátedra de Anatomía Vegetal, Facultad de Ciencias Naturales e Instituto Miguel Lillo, UNT, San Miguel de Tucumán 4000, Argentina; 3 Centro de Investigación en Biofísica Aplicada y Alimentos (CIBAAL), CONICET-UNSE, Santiago del Estero 4206, Argentina; 4 Instituto de Física del Noroeste Argentino (INFINOA), CONICET-UNT, Facultad de Ciencias Exactas y Tecnología, FACET, UNT, San Miguel de Tucumán 4002, Argentina

**Keywords:** silver nanoparticles, cupric oxide nanoparticles, *Penicillium digitatum*, *Penicillium italicum*, *Geotrichum citri-aurantii*

## Abstract

Postharvest fungal diseases are the main cause of economic losses in lemon production. The continued use of synthetic fungicides to control the diseases favors the emergence of resistant strains, which encourages the search for alternatives. The aim of this study was to assess the efficacy of metallic nanoparticles (NPs) as antifungal agents against local isolates of *Penicillium digitatum* and *Penicillium italicum*, each of them in a fungicide-sensitive and -resistant version, and a *Geotrichum citri-aurantii* isolate. NPs of ZnO, CuO, and Ag were synthesized and characterized by spectroscopy and microscopy, presenting average sizes < 25 nm and spherical shapes. ZnO-NPs did not present antifungal activity at the assayed conditions, while the minimum fungicidal concentrations (MFCs) were 1000 and 10 µg mL^−1^ for CuO-NPs and Ag-NPs, respectively. The NPs’ antimicrobial action included conidial membrane permeability and strong intracellular disorganization. Moreover, the Ag-NPs reduced green mold incidence on inoculated lemons when applied to the fruit. Taken together, Ag-NPs were effective in inhibiting both fungicide-sensitive and -resistant isolates of the main lemon postharvest pathogens, suggesting their potential use as an alternative approach.

## 1. Introduction

Significant economic losses worldwide are generated due to citrus postharvest fungal diseases, which include green and blue molds and sour rot, caused by *Penicillium digitatum*, *Penicillium italicum*, and *Geotrichum citri-aurantii*, respectively [1,2,3]. Argentina is the world’s sixth largest producer of citrus fruits, and our region, located in the northwest of Argentina, accounts for around 80% of the country’s lemon production [4]. Currently, to control these fungal diseases, a reduced spectrum of fungicides can be applied, such as thiabendazole (TBZ), imazalil (IMZ), and pyrimethanil (PYR). However, their continued application results in the emergence of resistant pathogenic strains [5,6] and serious risks to human health and the environment [7]. Thus, there is an increasing interest in searching for new strategies to control phytopathogens [8,9] in all fresh fruit-producing areas.

Nowadays, the use of nanoparticles (NPs) has appeared as a promising tool to apply in diverse fields as medicine, electronics, energy, and agriculture [10]. However, the use of nanotechnology in the agricultural sector has raised concerns about its potential environmental and health risks [11,12]. Nanoparticles (10–100 nm, in one dimension at least) could be synthesized by different processes, including physical, chemical, and biological methods [13], and offer significant advantages, particularly as antimicrobial agents. One of them includes a high surface-to-volume ratio that enhances interaction with the microorganisms [14]. Several reports have demonstrated the antimicrobial action of NPs containing metal ions such as zinc, silver, and copper [15,16,17,18]. In this regard, the action of ZnO-, CuO- and Ag-NPs against several fungi that affect diverse crops, such as *Fusarium solani*, *Botrytis cinerea*, *Alternaria alternata*, *Monilinia fructicola*, *Colletotrichum gloeosporioides*, *F. oxysporum*, and *Verticillium dahliae* [19,20], has been of reported. Even though the antifungal effect of selenium-containing nanocomposites on citrus pathogens such as *Penicillium digitatum* has been recently studied [21,22], the use of metallic NPs on these phytopathogens has not been explored. Therefore, the aims of this study were to synthesize the NPs of ZnO, CuO, and Ag and to evaluate their antifungal action against local isolates such as *G. citri-aurantii* and both fungicide-sensitive and -resistant *Penicillium* spp.

## 2. Materials and Methods

### 2.1. Chemicals

ZnC_4_H_6_O_4_ (Bernangel, Buenos Aires, Argentina); NaOH, Isopropanol, Ethylene Glycol (EG), and H_2_O_2_ (Cicarelli, Santa Fe, Argentina); CuSO_4_, AgNO_3_ (Anedra de Research Ag, Buenos Aires, Argentina); KOH (Merck, Darmstadt, Germany); Polyvinylpyrrolidone (PVP K30), Tween 80, and Dichlorofluorescein diacetate (H_2_DCFDA) (Sigma-Aldrich, Chemical Co., St. Louis, MO, USA); SYTOX™ Green nucleic acid stain (Thermo Fisher Scientific Inc., Waltham, MA, USA). Other chemicals used were of analytical grade.

### 2.2. Synthesis of Zinc Oxide Nanoparticles

For the synthesis of ZnO-NPs, the protocol reported by Sandoval et al. [23] was followed. This process involves a low-temperature reaction (ice bath) using 230 mL of 0.0012 M zinc acetate and 20 mL of 0.02 M NaOH, both in isopropanol. Subsequently, this mix was heated to 65 °C for 2 h, resulting in the formation of a transparent suspension of NPs. Finally, isopropanol was removed through evaporation at 65 °C under a vacuum pressure of 400 mmHg to obtain fine powder. Different concentration suspensions were prepared by resuspending the powder in isopropanol.

### 2.3. Synthesis of Cupric Oxide Nanoparticles

A green synthesis approach was carried out for the preparation of CuO-NPs [24], using Aloe Vera (AV) extract. Five leaves were cleaned with distilled water, fragmented into small pieces, and dried in an oven set at 80 °C for 24 h. To obtain AV extract, 10 g of dried material was pulverized using a pestle, resuspended in 100 mL water, and boiled for 20 min. For the synthesis of CuO-NPs, 10 mL of 0.3 M CuSO_4_ reacted with 50 mL of 10% AV extract at 100 °C for 1 h, with the formation of a black precipitate. Subsequently, the product was dried in an oven at 80 °C and underwent a sintering procedure at 300 °C for 30 min in muffle to improve size and form. The resulting NPs were maintained in darkness. To prepare the work suspension, CuO-NPs were resuspended in water and sonicated to obtain a dispersed suspension.

### 2.4. Synthesis of Silver Nanoparticles

Silver nanoparticles were synthesized according to Leng et al. [25] with some modification, using polyvinylpyrrolidone as a stabilizer. In a typical synthesis procedure, 35 mL of ethylene glycol (EG) was heated to 160 °C at agitation speed of 800 rpm. Then, 15 mL of EG containing 0.65 M silver nitrate and 1.6 g polyvinylpyrrolidone was gradually added to the stirring solution, maintaining an addition rate of 1 mL min^−1^. Upon completion of the reduction reaction, the resulting mixture was cooled to room temperature. Finally, the Ag-NPs were separated from EG by a dilution process with distilled water and three rounds of centrifugation at 15,800× *g* for 20 min each to obtain a Ag-NPs suspension in water.

### 2.5. Characterization of Metallic Nanoparticles

The ZnO-, CuO- and Ag-NPs obtained were characterized by UV–visible spectroscopy (Beckman DU7500 UV-VIS Diode array spectrometer, Spectralab Scientific Inc., Markham, ON, Canada). The form and size were determined using transmission electron microscopy (TEM) (Zeiss-Leo 906E, Zeiss, Oberkochen, Germany) and scanning electron microscopy (SEM) (Zeiss-Supra 55VP, Zeiss, Oberkochen, Germany). Elemental composition of NPs was determined using energy-dispersive spectroscopy (EDS), with an Inca Penta FET X3 instrument, Oxford (CIME-CONICET-UNT, Tucumán, Argentina), and X-ray diffraction (XRD), performed using an X-ray diffractometer (Rigaku Miniflex 300, Rigaku, Tokyo, Japan) operated at 30 kV and 100 mA, utilizing CuKα radiation with a wavelength of 1.5406 Å within the 2θ angle range of 20° to 80°. The hydrodynamic diameter of NPs was determined using dynamic light scattering (DLS) and the Zeta potential (ξ) value was determined using a Horiba SZ-100 analyzer (Horiba Ltd., Kyoto, Japan). DLS measurements were conducted at a detection angle of 173°, and 100 fixed runs were performed for each sample, with a duration of 30 s per run. Data analysis was performed on Horiba NextGen Project SZ-100 (P2000447001J 2.00) to obtain the hydrodynamic radius and charges of the NPs.

### 2.6. Fungal Isolates and Conidial Suspensions Preparation

For the evaluations of lemon phytopathogens, fungal isolates previously characterized from the INSIBIO (CONICET-UNT) laboratory collection were used. These included fungicide-sensitive variants of *P. digitatum* (PDS, F-Pd07-S) and *P. italicum* (PIS, F-Pi09-S), and fungicide-resistant variants to imazalil, thiabendazole, and pyrimethanil from *P. digitatum* (PDR, F-Pd17-R31) and *P. italicum* (PIR, F-Pi15-R29) [26]. Additionally, a *G. citri-aurantii* (GC, F-Gc-S) isolate was included. For the preparation of the conidial suspension, the fungi were allowed to grow on potato dextrose agar (PDA) for 5 d at 22 ± 1 °C. Sterile distilled water containing 0.05% Tween 80 (Sigma-Aldrich) was used to scrape the colony surface, and the conidia were collected and filtered through two layers of cheesecloth to remove hyphal fragments. The cellular concentration was adjusted with water to 1 × 10^6^ conidia mL^−1^ following counting in a Neubauer chamber [27].

### 2.7. Fruit

Lemons (*Citrus limon* (L.) Burm. F., cv. Eureka) were harvested at Las Tipas farm (Citrus Fruits S.R.L) from Tucumán, Argentina (26°37′60.0″ S 65°25′00.0″ W) and were free from any postharvest treatment or coating. Before treatments, each lemon was superficially disinfected (70% ethanol), rinsed with tap water, and allowed to air-dry at room temperature.

### 2.8. Determination of Conidia Growth and Minimum Fungicidal Concentration

Different approaches were used to assess the antifungal effect of NPs against the phytopathogenic isolates. In the case of ZnO-NPs, a test in potato dextrose liquid medium was conducted, covering a concentration range of up to 5000 μg mL^−1^. The effect of CuO-NPs and Ag was first assessed through a semiquantitative assay. This analysis was conducted by exposing conidial suspensions to different concentrations of CuO-NPs (from 100 to 1000 μg mL^−1^) and Ag-NPs (from 1 to 10 μg mL^−1^) for 24 h. Subsequently, a 5 μL aliquot of treated suspensions was spotted onto PDA medium using serial dilutions (undiluted, 1/100, and 1/1000). On the other hand, viability was quantified in terms of colony-forming units per milliliter (CFU mL^−1^) after exposure of the conidia to different concentrations of CuO-NPs or Ag for 24 h. Colonies were counted after 4 d of incubation at 22 ± 1 °C. The minimum fungicidal concentration (MFC) is defined as the lowest concentration of NPs resulting in the death of 99.9% of the inoculum [28].

### 2.9. Determination of Infective Capacity of Treated Conidia

To assess the residual infectivity on the conidia of lemons after treatment with the NPs, a method described by Cerioni et al. [27] was followed, with some modifications. The artificial inoculation was performed using a steel rod previously immersed in control or treated conidial suspensions by exposing conidia to different concentrations of CuO- or Ag-NPs for 24 h. The rod tip was 1 mm wide and 2 mm long, which penetrated both flavedo and albedo tissues but not juice sacs of lemons. Fruit was stored at 20 °C and 95% relative humidity for 5 d, and incidence of infection was recorded at inoculation sites. The asymptomatic fruit was stored for 14 d before being discharged.

### 2.10. Evaluation of Conidial Membrane Integrity

To determine the impact of NPs on conidial membrane integrity, the fluorescent probe SYTOX^TM^ Green (Thermo Fisher, Buenos Aires, Argentina) was used, following the protocol described by Olmedo et al. [29] Conidial suspensions were treated at MFC or sublethal concentration for each NP for 24 h and then were washed and incubated with SYTOX^TM^ Green (5 µM) for 30 min in darkness. Conidia were visualized under a fluorescence microscope Olympus IX51 equipped with an Olympus digital camera, QColor5 (Q-imaging). The fluorescence emission was examined and photographed using a filter set of 450–490 nm for excitation and 515–565 nm for emission. Three replicates were performed for each condition, and the assay was repeated three times.

### 2.11. Quantification of Reactive Oxygen Species Accumulation on Conidia

To determine the accumulation of reactive oxygen species (ROS) after treatments with NPs, a protocol reported by Cerioni et al. [30] was followed, with some modifications. Conidial suspensions were treated as described above, washed, and incubated with 10 μM H_2_DCFDA probe in darkness overnight. After removing the probe, the fluorescence intensity was measured using an ISS-PCI spectrofluorometer (Champaign, IL, USA) and normalized with respect to the initial conidia concentration. Conidia treated with 600 mM H_2_O_2_ was included as positive control of ROS formation.

### 2.12. Evaluation of Conidia Ultrastructure

Conidia treated for 24 h with metallic NPs at the MFC or water (controls) were ultrastructural characterized through transmission electron microscopy (TEM). Conidia were processed as described by Cerioni et al. [30]. Observations were made using a Hitachi HT7800 (Hitachi, Chiyoda City, Japan) transmission electron microscope, available at the Comprehensive Center for Electron Microscopy (CME, CONICET-UNC, Córdoba, Argentina).

### 2.13. Application of Ag-NPs on Lemons

The Ag-NPs were evaluated by in situ application on lemons wounded using a stainless steel rod previously immersed into a freshly prepared conidial suspension of *P. digitatum* (PDS and PDR). After inoculation, the lemons were allowed to dry for 30 min at room temperature. Subsequently, 10 µL of Ag-NPs at concentrations of 500 and 1000 µg mL^−1^ were directly applied to the wounds. As a control, 10 µL of water was added to the wounds. The lemons were incubated for 5 d in a chamber with controlled conditions of temperature (20 °C) and relative humidity (95%). The results were obtained by evaluating the incidence of the disease.

### 2.14. Statistical Analysis

For the in vitro assays, three complete sets of experiments were conducted, encompassing three repetitions for each condition. The in vivo assays comprised two repetitions involving 10 lemons (each with two wounds) for each condition. In all cases, the data underwent variance analysis, followed by Tukey’s test using Infostat software v 2020I [31]. Differences of *p* ≤ 0.05 were considered significant.

## 3. Results

### 3.1. Synthesis and Characterization of Nanoparticles

**Figure 1:**
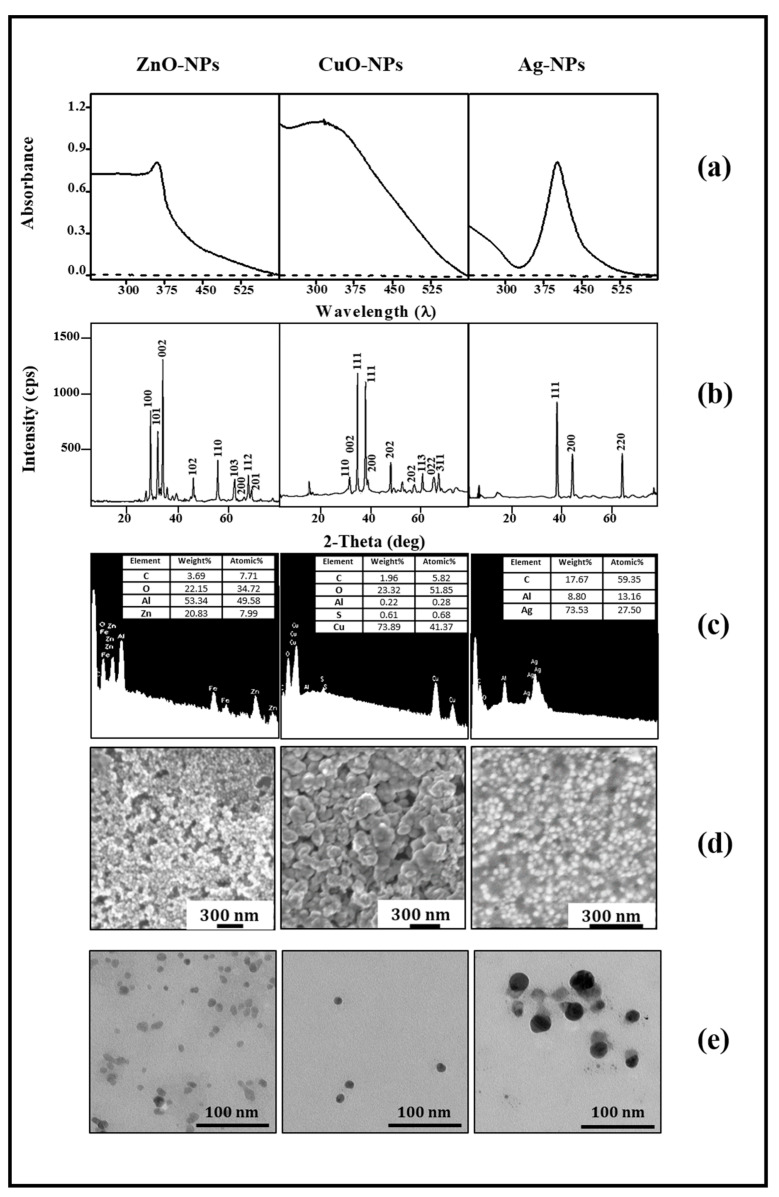
Characterization of nanoparticles by spectroscopic techniques and electron microscopy. (**a**) UV-VIS spectra of NPs resuspended in isopropanol (ZnO-NPs) or water (CuO-and AgNPs) (solid line), and solvent reference baseline (dotted line); (**b**) X-ray spectra (XRD); (**c**) energy-dispersive spectra (EDS); (**d**) scanning electron microscopy (SEM); (**e**) transmission electron microscopy (TEM). As indicated, the left panels show ZnO-NPs, the central panels CuO-NPs, and the right panels Ag-NPs. Bars in the electron microscopy images indicate the scale.

Three types of metallic NPs (ZnO-, CuO-, and Ag-NPs) were synthesized, following methods described in the Materials and Methods section, and were subjected to characterization through various analytical techniques, as shown in Figure 1. The analysis involved UV-VIS spectroscopy from 230 to 600 nm of the NP preparations resuspended on the solvents used for subsequent in vitro evaluation, either isopropanol for ZnO-NPs or water for the other NPs (Figure 1a). In the case of ZnO-NPs, the spectrum exhibited absorption from wavelengths in the UV range up to nearly 360 nm, followed by a sharp decrease in the absorbance. For CuO-NPs, an absorbance peak was observed at 310 nm, while in the case of Ag-NPs, a surface plasmon resonance signal was detected at 406 nm. As a reference to the baseline, the spectra of solvents were included. An X-ray powder diffraction analysis was carried out to obtain information on the elemental composition of the NPs (Figure 1b). In the case of ZnO-NPs, precise correlations were found with the Bragg planes (100), (101), (002), (102), (110), (103), (200), (112), and (201). For CuO-NPs, the following peaks were identified: (110), (002), (111), (111), (200), (202), (202), (112), (022), and (311). The X-ray diffraction pattern for the Ag-NPs presented peaks at (111), (200), and (220). Thus, distinctive diffraction peaks were observed for each type of NP, corresponding to a precise correlation between the peaks and their respective materials. Furthermore, the composition of the NPs was confirmed by energy-dispersive X-ray spectroscopy (EDS) (Figure 1c). For ZnO-NPs, significant proportions of O_2_ (22.15%) and Zn (20.83%) were identified, confirming the ZnO nature of the NP. The EDS analysis of CuO-NPs revealed that Cu (73.89%) and O_2_ (23.32%) were the predominant elements, unequivocally demonstrating the CuO nature of the sample. The elemental composition of the Ag-NPs revealed a Ag content of 73.53%, confirming its metallic nature, with a significant presence of C (17.67%) attributed to the stabilizer (PVP). NP morphology was assessed using scanning electron and transmission electron microscopies (SEM and TEM) (Figure 1d and Figure 1e, respectively), with most NPs displaying spherical shapes. TEM also provided the calculated NP average sizes: 11 ± 0.6 nm for ZnO-NPs, 11 ± 0.5 nm for CuO-NPs, and 25 ± 2.6 nm for Ag-NPs. Additionally, a dynamic light scattering (DLS) analysis was performed to assess the surface charge and to determine the hydrodynamic radius of the NPs. The zeta potential values for the NPs were 20.0 mV for ZnO-NPs, −39.8 mV for CuO-NPs, and −41.8 mV for Ag-NPs. The hydrodynamic radius of ZnO-NPs was not determined due to the presence of the organic solvent, while CuO-NPs and Ag-NPs show hydrodynamic radii of approximately 9 nm and 82 nm, respectively.

### 3.2. Antifungal Action of Nanoparticles

**Figure 2:**
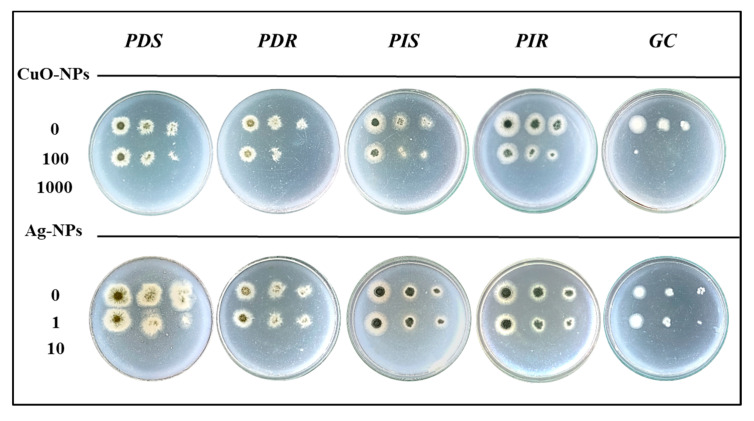
Antifungal activity of nanoparticles. Conidial suspensions (10^6^ CFU mL^−1^) of the indicated isolates were exposed for 24 h to different NP concentrations (μg mL^−1^). After treatments, serial dilutions of the cells were plated on PDA and grown for 4 d at 22 ± 1 °C. Controls corresponding to cells incubated without NPs were included. The growth of conidia treated with CuO-NPs (**upper panel**) and Ag-NPs (**lower panel**) is shown as representative images from three independent experiments. The last point of each row contains a 1/1000 dilution of the original culture.

The antifungal activity of metallic NPs against the fungicide-sensitive or -resistant phytopathogens was evaluated. ZnO-NPs did not present antifungal activity at analyzed concentrations up to 5000 µg mL^−1^. Figure 2 shows a semiquantitative assessment of conidial growth after 24-h exposure to CuO- or Ag-NPs. In control samples, characteristic colonies for each pathogen were observed whose radial growth decreased as the conidial dilution increased, as expected. The colony growth of all pathogens was inhibited when conidia were pre-treated with a concentration of 1000 µg mL^−1^ for NPs-CuO and at 10 µg mL^−1^ for NPs-Ag. At concentrations that are 10-fold lower, a decrease in radial growth compared to untreated controls was observed, indicating sublethal conditions in which only part of the conidia remained viable. Therefore, colony-forming units per milliliter (CFU mL^−1^) were determined for quantification (Table 1). At a sublethal concentration of 100 µg mL^−1^ of CuO-NPs, a 62% inhibition was observed in the PDS population, which represents a decrease in an order of magnitude in the values of CFU mL^−1^, from 1.1 × 10^6^ to 2.0 × 10^5^. Additionally, an 84% inhibition was observed for PDR, with a decrease to 9.5 × 10^4^ CFU mL^−1^. The viability inhibition for PIS, PIR, and GC isolates was close to 90%. For Ag-NPs, at a sublethal concentration (1 μg mL^−1^), the range of inhibition varied between 95 and 99% for all the evaluated fungi, except for PDR with a 75% reduction in conidia viability. The minimum fungicide concentration (MFC) was 1000 μg mL^−1^ for CuO-NPs and 10 μg mL^−1^ for Ag-NPs for all phytopathogens evaluated.

### 3.3. Residual Infection of Conidia Treated with Nanoparticles

**Figure 3:**
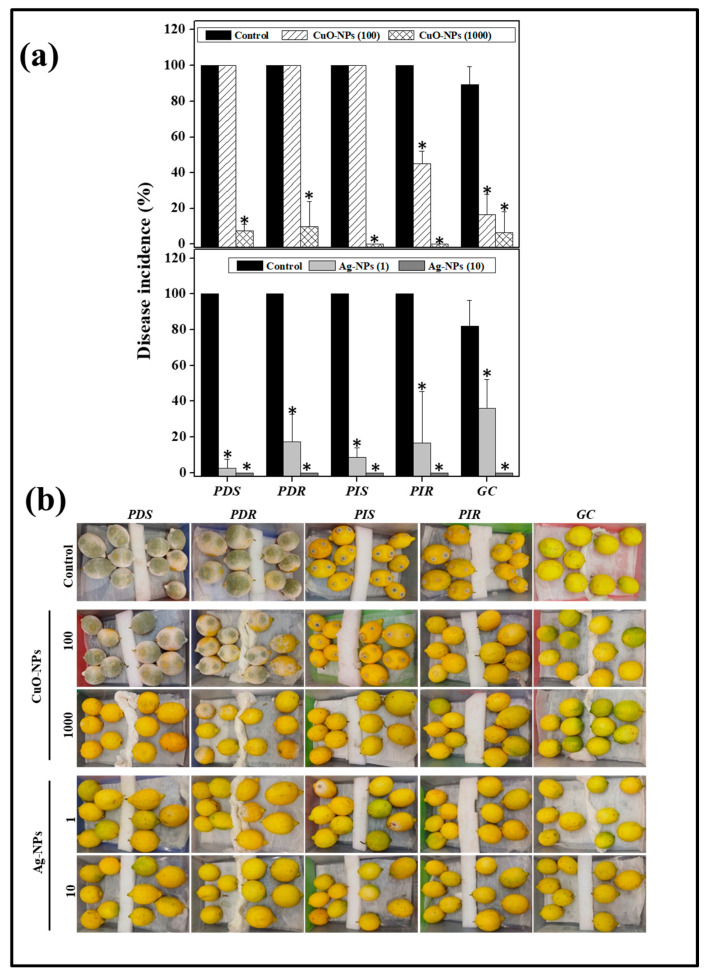
Effect of nanoparticles on infectivity capacity of conidia on lemons. (**a**) Lemons were inoculated using conidia treated with the indicated NP concentrations (μg mL^−1^) and the incidence of the disease was evaluated as a percentage of the corresponding control. The results were averaged from two experiments, using 10 lemons and 2 wounds in each case. Asterisks (*) indicate significant differences with respect to the control group according to Tukey’s analysis, with a *p*-value ≤ 0.05 (**b**) Representative images of disease incidence for the specified isolate and conditions.

The residual infectivity of conidia treated for a 24 h period with CuO- and Ag-NPs was evaluated on Eureka lemons (Figure 3a,b). All the fruit inoculated with untreated conidia exhibited high disease incidence at 5 d of incubation. When conidia were treated with CuO-NPs at a sublethal concentration (100 μg mL^−1^), PDS, PDR, and PIS registered a similar incidence to controls, while a reduction of nearly 70% was achieved for GC, being the most sensitive of the tested fungi. On the other hand, when conidia were treated with Ag-NPs at a sublethal concentration (1 µg mL^−1^), a significant decrease in disease incidence was achieved for all the pathogens, with inhibitions between 70 and 95%. As was expected, when the conidia were treated either with CuO-NPs or Ag-NPs at the MFC, no development of the postharvest diseases was observed until 14 d incubation.

### 3.4. Effect on Conidial Membrane Integrity After Nanoparticle Treatments

**Figure 4:**
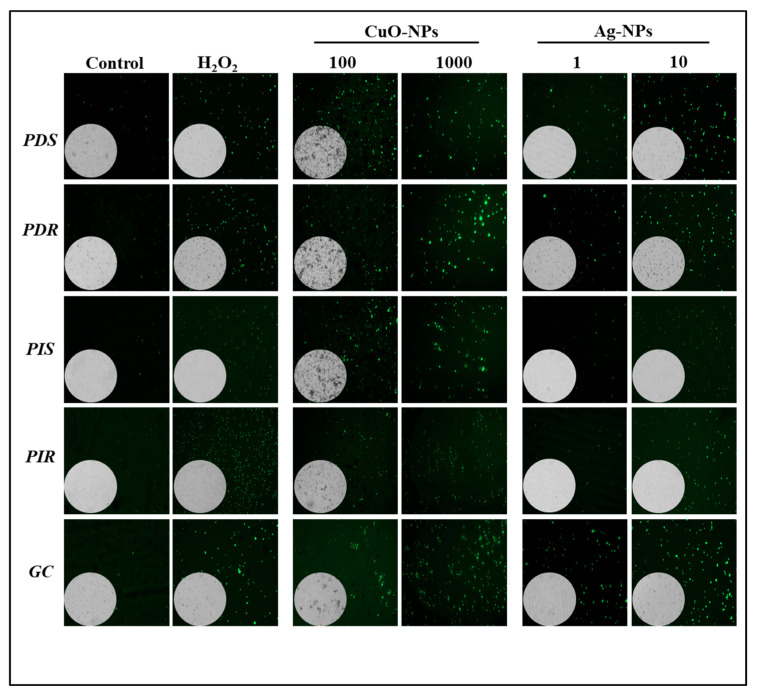
Effect of nanoparticles on membrane integrity. Conidial suspensions of indicated isolates were treated for 24 h with the indicated concentration of NPs (µg mL^−1^) and then exposed to 0.5 µM SYTOX^TM^ Green for 30 min. Fluorescent field images at a 40× magnification accompanied by a bright field (insets) are shown. The left panels display negative and positive controls, corresponding to conidia treated either with water or 600 mM H_2_O_2_, respectively. The images are representative of three independent experiments.

In order to analyze the effect of NPs on fungal conidia, a study of the cell membrane integrity was carried out using the SYTOX^TM^ Green probe (Figure 4). In the dark field, fluorescent conidia were observed for all fungal isolates treated in the presence of CuO- and Ag-NPs, indicating increases in membrane permeability in a concentration-dependent manner. As the positive control of membrane damage, a treatment with 600 mM H_2_O_2_ was included, which shows fluorescence for all the phytopathogens, similar to NP treatments. No fluorescence emission was observed in samples incubated with water. It should be noted that most of the conidia were clearly visible in the bright field, except at the maximum CuO-NP concentration due to dark sediment being present.

### 3.5. Production of Reactive Oxygen Species After Nanoparticle Treatments

**Figure 5:**
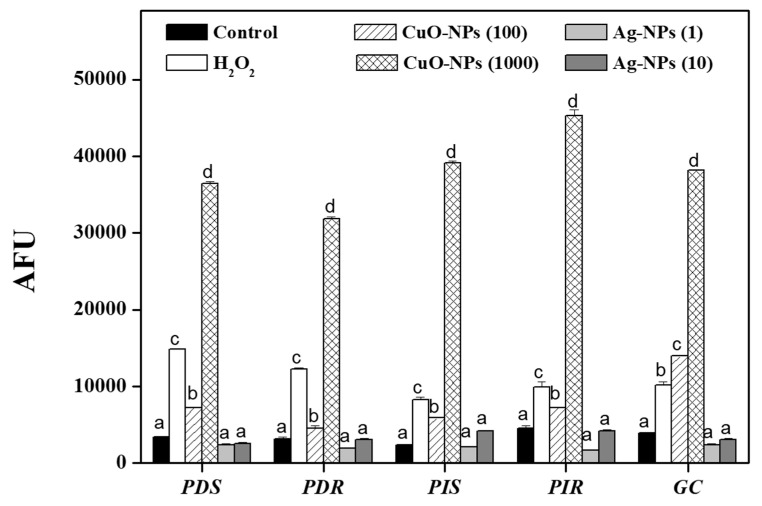
Effect of nanoparticles on ROS generation. After exposing conidia to the indicated concentrations of CuO- and Ag-NPs (μg mL^−1^) for 24 h, the amount of intracellular ROS was determined. The results are expressed as the mean ± standard deviation from three independent experiments. Different letters indicate significant differences between treatments according to Tukey’s analysis, with a *p*-value ≤ 0.05.

A proposed mechanism for the action of NPs on microorganisms is the induction of ROS generation. Figure 5 shows the quantitative evaluation of ROS accumulation in treated conidia using the H_2_DCFDA probe. At lethal and sublethal concentrations of CuO-NPs, a significant increase in ROS accumulation in the conidia was observed, with respect to the control, being 10-fold higher at the lethal concentration. Nevertheless, ROS production was not detected after 24 h of treatment with both Ag-NP concentrations in any of the evaluated pathogens.

### 3.6. Conidial Internal Structure After Nanoparticle Treatments

**Figure 6:**
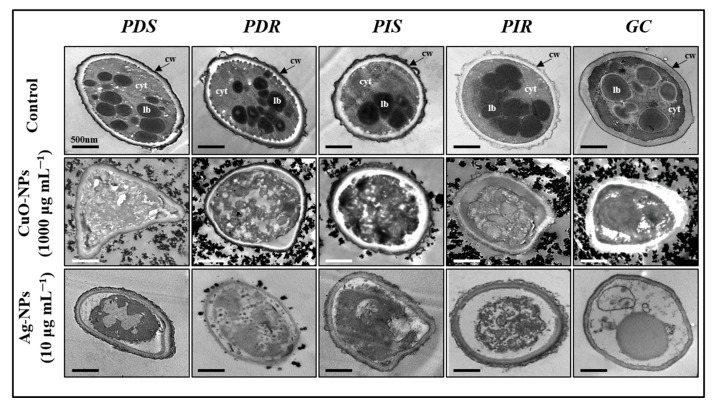
Intracellular damage induced by nanoparticles. Conidial suspensions were exposed to the minimum fungicidal concentration of NPs for 24 h and then visualized by transmission electron microscopy (TEM). The tests were repeated three times for each treatment, and at least two replicates were examined. The top panels show control conditions for each isolate, while the remaining panels display conidia treated with the indicated nanoparticles. In micrographs, a scale bar of 500 nm is represented. cw, cell wall; cyt, cytoplasm; lb, lipid body.

The ultrastructure of the conidia was examined using transmission electron microscopy (TEM) (Figure 6). All evaluated fungi in control condition revealed an appropriate organization of the cell walls and the cytoplasm and the presence of some internal structures as lipid bodies, indicating an overall undisturbed structure.

However, after exposing the conidia to the NPs at the MFC for 24 h, notable cellular damage was evident, including cell deformation and clear cytoplasmic disorganization, with the presence of intracellular cavities and vesicles. Significant modifications in the membrane and cell wall structure occurred, characterized by contraction and irregularities. Taken together, these results indicate irreversible cellular damage after treatment with both NPs.

### 3.7. Green Mold Incidence After In Situ Application of Ag-NPs on Lemons

**Figure 7:**
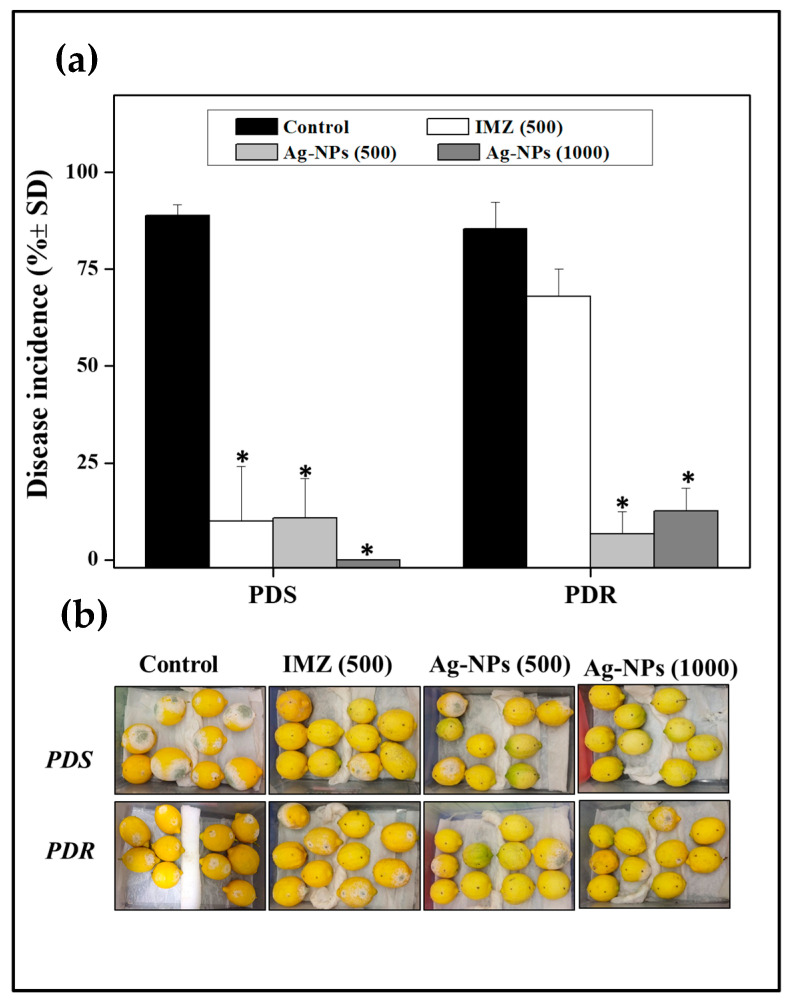
Disease incidence of green mold caused by sensitive and resistant strains. (**a**). The fruits were inoculated with a suspension of 1 × 10^5^ CFU mL^−1^ of the indicated strains and treated with 10 μL of NP suspensions at the indicated concentration, imazalil (commercial fungicide), or water (control). Green mold incidence was assessed as a percentage. Asterisks (*) indicate significant differences compared to the control group according to Tukey’s test, with a *p*-value ≤ 0.05. (**b**). Representative images of the treatment with Ag-NPs are shown.

Considering the higher in vitro efficacy of silver NPs against the tested fungi compared to the other NPs, the antifungal action of Ag-NPs was evaluated in situ on artificially inoculated lemons. In the control group, an elevated disease incidence was observed (around 90%) on inoculated fruit (Figure 7a,b). As expected, the application of IMZ at 500 µg mL^−1^ showed differential results depending on the assayed fungal isolate. Indeed, IMZ was able to control green mold caused by fungicide-sensitive isolate, while it allowed a disease incidence nearly 65% for the resistant isolate. In contrast, the application of Ag-NPs demonstrated significant antifungal action against both *P. digitatum* isolates, presenting disease incidences below 10%.

## 4. Discussion

In this study, zinc oxide, cupric oxide, and silver NPs were synthesized, characterized, and applied to inhibit the growth of fungicide-sensitive or -resistant citrus phytopathogens, both in vitro and on the lemon fruit.

In all cases, the synthetized metallic NPs presented characteristic UV-VIS spectra, containing absorbance peaks that were consistent with those previously reported in the literature for each NP [25,32,33]. The nature and elemental composition of them were confirmed by XRD and EDS techniques. In addition, considering previous analyses [34,35] and the obtained zeta potential values, it was expected that CuO and Ag-NPs would present greater dispersion and stability in the colloidal suspension than those of ZnO-NPs. The average size of CuO-NPs, after the sintering process that eliminated all organic material, was similar according to DLS and TEM determinations. However, only the size measured by TEM was taken into account for Ag-NPs, since the DLS values might be overestimated by the PVP organic layer and the surrounding aqueous solvation layer, as previously argued [36]. The present characterization indicated that the obtained NPs exhibit two important features: nanometric size and spherical shape. The spherical shape facilitates a uniform distribution of the NPs, preventing the formation of aggregates and maximizing their contact area with microorganisms [32,37]. Furthermore, nanometric particles, bearing a higher surface-to-volume ratio, tend to display high antimicrobial activity due to the increased interaction with the microbial cell envelopes [25].

The present results demonstrated that CuO- and Ag-NPs exhibited antifungal activity against all evaluated fungi, whereas ZnO-NPs did not show activity. It is possible that the zeta potential value of ZnO-NPs affects suspension stability, leading to the NP aggregation in the incubation medium. The inhibition of all pathogens was achieved at a concentration of 10 μg mL^−1^ Ag-NPs, while a 100 times larger concentration of CuO-NPs was required to obtain a similar deleterious effect. At present, there are few reports where NPs were evaluated against filamentous fungi. Previous reports have demonstrated similar results when comparing different NPs against *Alternaria alternata* and *Pyricularia oryzae*, where Ag- and CuO-NPs were effective, while ZnO-NPs did not significantly inhibit mycelial growth compared to the control [38]. Malandrakis et al. [20] have evaluated the sensitivity of several fungi to both CuO- and Ag-NPs, showing a moderate inhibition only against *B. cinerea* at 307 μg mL^−1^ Ag-NPs. In another report, 150 µg mL^−1^ Ag-NPs were able to inhibit nearly 50% of the *P. verrucosum* population [39]. Note that in these reports, the Ag-NP concentration necessary to achieve the inhibitory effect was between 15- and 30-fold higher than that used in our conditions. In addition, Shammout and Awwad [40] reported *Aspergillus niger* growth inhibition using 5000 μg mL^−1^ CuO-NPs, showing more tolerance with respect to the phytopathogenic fungi tested here. It is worth noting that the effect of NPs against fungicide-sensitive and -resistant isolates was comparable, which could allow the control of postharvest diseases caused by fungal-resistant isolates.

When comparing the conidial infective capacity on lemons using treated fungi, Ag-NPs were more effective than CuO-NPs in reducing the residual incidence, impairing the progress of diseases even at a sublethal concentration. In that condition (1 μg mL^−1^ of Ag-NPs), a decrease of nearly 96% in disease incidence was observed, although the colony-forming units (CFUs) counted (e.g., 10^4^ CFU mL^−1^ for PDS) provided enough inoculum for mold development on lemons, as it has been previously reported [41,42].

The CuO-NPs and Ag-NPs induced cell disorganization and membrane permeability on the evaluated phytopathogens. The mechanisms of action of NPs on bacteria involve multiple cellular targets [37,43]. The use of treatments that affect multiple targets could reduce fungal development, in contrast to fungicides with a single mode of action, such as imazalil, which are often evaded by resistant isolates [44,45]. Additionally, CuO-NPs caused an increase in the generation of ROS, similar to what has been reported for the oomycete *Phytophthora nicotianae* by Chen et al. [46]. Ag-NPs (average size 25 nm) at the evaluated concentrations did not lead to ROS production after 24 h of treatment. The difference in ROS production between Ag-NPs and CuO-NPs may be attributed to a variety of factors, such as size, concentration, chemical composition, and their interaction with the specific microorganisms. Despite this, it has been reported that Ag-NPs conduced to ROS accumulation in *Candida albicans* and *Fusarium graminearum* [47,48], supporting the present results. Carlson et al. [49] highlight the variability in ROS generation depending on nanoparticle size when acting over macrophages. They reported that 15 nm Ag-NPs generated ROS, while 30 nm particles did not, although both yielded positive results in cytotoxicity assays. Together, Ag-NPs cause severe ultrastructural damage and a strong inhibition of growth to all the citrus pathogens in the assayed conditions, despite the absence of ROS production.

Due to the best antifungal activity demonstrated to Ag-NPs, their efficacy to control green mold incidence was evaluated. This step was crucial to determine if the antifungal properties observed in vitro were maintained under conditions closer to practical applications. The obtained results show that the in situ application of 500 µg mL^−1^ Ag-NPs was effective in decreasing the incidence of green mold on lemons, including mold caused by the *Penicillium* spp. fungicide-resistant isolate. Previous reports have explored the effect of several nanoparticles to control infectious diseases in fruits. Iliger et al. [50] used CuO-NPs to control rot in chili peppers caused by *Colletotrichum capsici* using 1000 µg mL^−1^ of NPs. Saqib et al. [51] evaluated iron oxide nanoparticles in vivo on apricots infected with *Rhizopus stolonifer* and found that a concentration of 10,000 µg mL^−1^ of NPs was needed to control this mold. It should be noted that metallic NPs can be risky for human health, since it is well known that may cause damaging effects, such as oxidative stress, cytotoxicity, and inflammation [52,53,54]. In this context, further research is necessary to assess the safety and viability of NPs (or their immobilized forms) before large-scale implementation for fruits, packaging, and commercial tanks.

## 5. Conclusions

In the present study, the antifungal activity of three types of NPs were evaluated providing a detailed description of their synthesis and characterization. CuO- and Ag-NPs presented the ability to inhibit in vitro lemon fungal pathogens. It has been observed that both NPs exert their action through membrane permeabilization and intracellular damage, while CuO-NPs also induced the production of ROS. Moreover, Ag-NPs reduced the residual infectivity of conidia even at sublethal concentrations and were able to control green mold on artificially inoculated lemons, especially mold caused by a fungicide-resistant isolate. Taken together, our results indicate that Ag-NPs are a promising alternative antifungal nanomaterial for the preservation of fruits against postharvest fungal pathogens.

## Figures and Tables

**Table 1:** Effect of nanoparticles on phytopathogens conidia viability *.

Pathogens	Control	CuO-NPs (µg mL^−1^)		Ag-NPs (µg mL^−1^)	
	-	100	1000	1	10
	CFU mL^−1^				
PDS	1.1 × 10^6^ a	2.0 × 10^5^ b	6.5 × 10^1^ c	2.2 × 10^4^ b	0 b
PDR	9.0 × 10^5^ a	9.5 × 10^4^ b	9.6 × 10^1^ c	1.5 × 10^5^ b	0 b
PIS	9.5 × 10^5^ a	4.8 × 10^4^ b	1.7 × 10^1^ c	1.7 × 10^4^ b	0 b
PIR	1.2 × 10^6^ a	2.5 × 10^4^ b	1.0 × 10^1^ b	4.0 × 10^3^ b	0 b
GC	8.5 × 10^5^ a	1.0 × 10^2^ b	0 b	5.0 × 10^3^ b	0 b

* Conidial suspensions were exposed to NPs both at sublethal concentrations and MFC, as indicated. Samples treated only with water were used as controls. Different letters indicate significant differences comparing each pathogen to their respective control according to Tukey’s analysis with a *p*-value ≤ 0.01. PDS-, PDR-, PIS-, and PIR-sensitive and -resistant *P. digitatum* and *P. italicum*; GC: *G. citri-aurantii*.

## Data Availability

The original contributions presented in the study are included in the article, further inquiries can be directed to the corresponding authors.
